# Linking the demographic, socio-economic and oral health status to oral health-related quality of life of the sudanese older adults: a cross sectional study

**DOI:** 10.1186/s12903-023-03089-6

**Published:** 2023-06-08

**Authors:** Mayson Ahmed Salih, Raouf Wahab Ali, Elwalid Fadul Nasir

**Affiliations:** 1 Department of Prosthetic Dentistry, Faculty of Dentistry, University of Science and Technology, P.O. Box 30, Omdurman, Sudan; 2 Faculty of Dentistry, University of Science and Technology, P.O. Box 30, Omdurman, Sudan; 3 Preventive Dentistry Department, College of Dentistry, King Faisal University, Alahsa, KSA Saudi Arabia

**Keywords:** Oral health status, OHRQoL, GOHAI, Wilson and Cleary model, DMFT, Sudan

## Abstract

**Background:**

Oral Health-Related Quality of Life (OHRQoL) is an important measure of patients’ needs and progress. Identifying the links between clinical and non-clinical factors with OHRQoL in a specific population will facilitate the development of effective preventive strategies. The aim of the study was to assess the OHRQoL of Sudanese older adults, and to identify the possible relations between clinical and non-clinical predictors with OHRQoL using Wilson and Cleary model.

**Methods:**

This cross-sectional study was conducted among older adults attending the out-patient clinics in Khartoum State’s Health Care Centers, Sudan. OHRQoL was assessed using the Geriatric Oral Health Assessment Index (GOHAI). Two modifications of Wilson and Cleary’s conceptual model were tested using structural equations modeling including: oral health status, symptom status, perceived difficulty of chewing, oral health perceptions, and OHRQoL.

**Results:**

249 older adults participated in the study. Their mean age was 68.24 (± 6.7) years. The mean GOHAI score was 53.96 (± 6.31) and trouble biting/chewing was the most commonly reported negative impact. Wilson and Cleary models showed that pain, Perceived Difficulty Chewing (PDC), and Perceived Oral Health had a direct effect on OHRQoL. In model 1, age and gender had direct effects on oral health status, while education had direct effects on OHRQoL. In model 2, poor oral health status is associated indirectly with poor OHRQoL.

**Conclusions:**

The OHRQoL of the studied Sudanese older adults was relatively good. The study partially confirmed Wilson and Cleary model as Oral Health Status was related directly to PDC and indirectly to OHRQoL through functional status.

## Background

Clinical indicators of oral conditions failed to consider the functional and psychosocial aspects of oral health, which led to development of Oral Health-Related Quality of Life (OHRQoL) measures [1, 2]. It is important to assess both the clinical variables and the patients’ perceptions of health to have accurate data to promote health and disease prevention programs, and for the allocation of health resources [3]. OHRQoL addresses the person-centered perspective and concerns that are related to oral health, disease outcomes, treatment need and success [4]. A significant association between the impact of reduced number of teeth, poor oral health, masticatory ability, and dental care on OHRQoL among older adults was shown in many studies [5–7]. The relationships between sociodemographic factors and self-perceived oral health among older adults were also investigated [8, 9].

Researches on Health-Related Quality of Life (HRQoL) were moved from the traditional descriptive methods to models, so that the causal relationships among the components could be investigated and clarified [10]. A conceptual model is defined as a visual or written product that explains, either graphically or in narrative form, the main things to be studied, the key factors, concepts, or variables and the presumed relationships among them [11]. Wilson and Cleary [12] proposed a conceptual model of the direct and mediated pathways between clinical and non-clinical variables in relation to the HRQoL. It is based on five abstract concepts: biological/physiological, symptom status, functional status, general health and quality of life, plus mentioning of individual and environmental factors. The Wilson and Cleary model was used to link xerostomia, edentulism, and prosthodontic treatment need to the OHRQoL among older adults [9, 13, 14].

The need for oral health care services is increasing worldwide as the number of older adults is growing, and as they retain more natural teeth [15]. In developing countries, there is a sizeable gap between resources allocated and the increasing population’s oral health needs [16]. Therefore, theoretically driven researches are important to facilitate a greater understanding of the dynamics of an individual’s experiences of oral health and, in turn, how oral health influences longer-term systemic health and well-being. The aim of this study was to assess the OHRQoL of older adults attending public health care centers in Khartoum state, Sudan using the Geriatric Oral Health Assessment Index (GOHAI), and to identify the possible relations between oral health status, demographic and socioeconomic predictors and OHRQoL using Wilson and Cleary model.

## Methods

### Study design and sampling procedure

This cross-sectional study was part of a research project that investigated the oral health status, oral functions, as well as the OHRQoL of older adults in Khartoum state, Sudan. The study was conducted at the outpatient clinics in Health Care Centers, Khartoum State, Sudan between 2018 and 2019. The sample size was calculated using the formula: (*n* = [DEFF*Np(1-p)]/ [(d^2^/Z^2^_1-α/2_*(N-1) + p*(1-p)]) in 5% precision at 95% CI ,1.5 as design effect and 87.7% prevalence of dental caries among Sudanese adults [17]. A sample of 249 participants was proportionally distributed among the seven localities of Khartoum state per the population as follow 12.1% [30] from Khartoum, 17.7% (44) from Jabal Aulia, 11.6% [29] from Bahri, 16.5% (41) from Sharg En Nile, 9.6% [24] from Omdurman, 18.9% (47) from Umm Bdda and 13.6% (34) from Karary. Two health care centers from each locality were selected using simple random sampling. All individuals who were ≥ 60-year-old attended the selected health care centers were invited to participate in the study fulfilling the desired number from each center. Participants were included in the study if they were permanent residents (resided for the last 5 years) in Khartoum state, Sudan, and were excluded if they were complaining from acute pain, traumatic injuries or oral conditions that may prevent communication or examination.  Sample size, sampling methods and techniques were described in an earlier paper [18]. Ethical approvals were obtained from the Ethical Committee of the University of Science and Technology (UST), Omdurman, Sudan (NO: UST/FD/REC: 20/8/ 2018) and the Management of Innovation, Development and Scientific Research, Ministry of Health, Khartoum State. All participants signed an informed consent form before the data collection.

### Data collection

Data were collected by interviewing the participants and conducting oral examination. The data were entered in Wilson and Cleary model to investigate the effect on oral health status, demographic and socio-economic factors on OHRQoL.

Four research team members were responsible of data collection (2 interviewed the participants and 2 performed clinical examination). Duplicate interview and examination of a total of 10% of the study sample was done at the beginning, mid-way and at the end of the data collection to confirm intra-examiner reliability. The intra-class correlation coefficient (ICC) was used to measure inter and intra-examiner reliability for GOHAI score and DMFT. Inter and intra-examiner reliability for GOHAI score and DMFT were 0.98 (95% CI 0.92–0.96) and 0.98 (95% CI 0.76–0.99), 0.92 (95% CI 0.44–0.99) and 0.99 (95% CI 0.86–0.99), 0.92 (95% CI 0.57–0.99) and 0.94 (95% CI 0.33–0.98) at the beginning, mid-way and at the end of the study, respectively.

### Oral health related quality of life

OHRQoL was evaluated using a validated Arabic version of the GOHAI-Ar questionnaire [19]. The GOHAI is composed of 12-items that reflects problems affecting older adults in 3 dimensions: physical functions, psychosocial functions, and pain and discomfort. Responses to GOHAI questions ranged from 1 = very often to 5 = never with a sum score between 12 and 60 corresponding to worst and best OHRQoL respectively. The participant’s responses to the GOHAI were analysed in three ways. (1) The responses to each question were coded as no impact (hardly ever and never) and negative impact (very often, fairly often and occasionally). (2) The domains of OHRQoL, a negative impact on the domain was reported when a participant reported a negative impact on one or more question constituting the domain. (3) The total score of GOHAI presented as a mean (± SD).

### Wilson and Cleary model

**Fig. 1:**
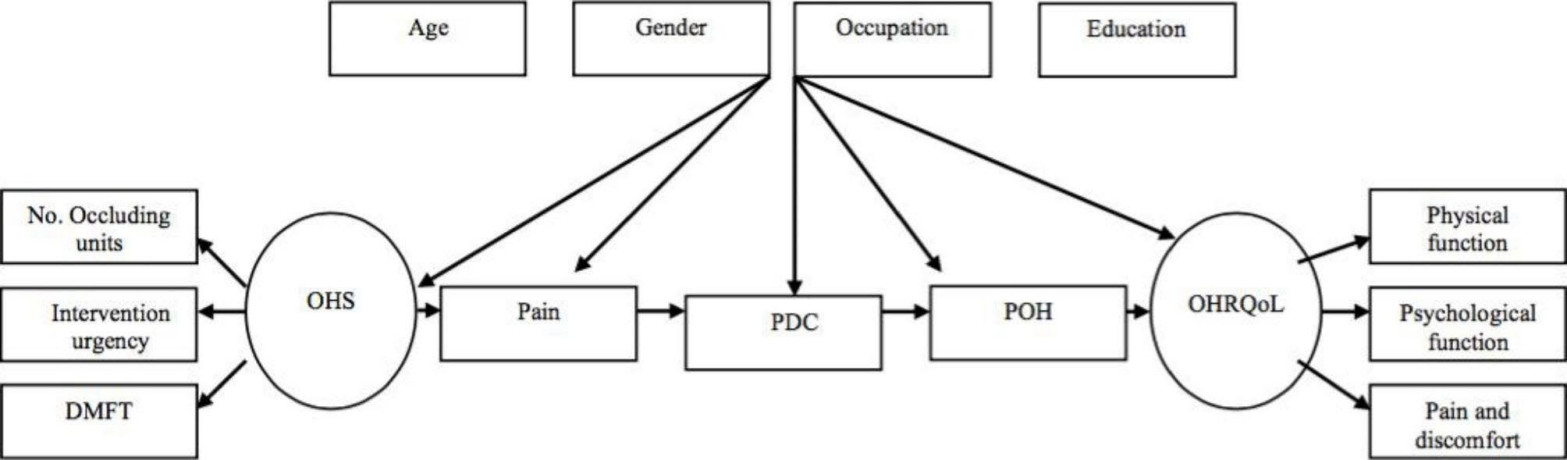
Wilson and Cleary model 1 examining the relationship between adjacent variables and demographic and socioeconomic characteristics in older adults. Residual errors were eliminated. *OHS: Oral Health Status, PDC: Perceived Difficulty of Chewing, POH: Perceived Oral Health, OHRQoL: Oral Health-Related Quality of Life

**Fig. 2:**
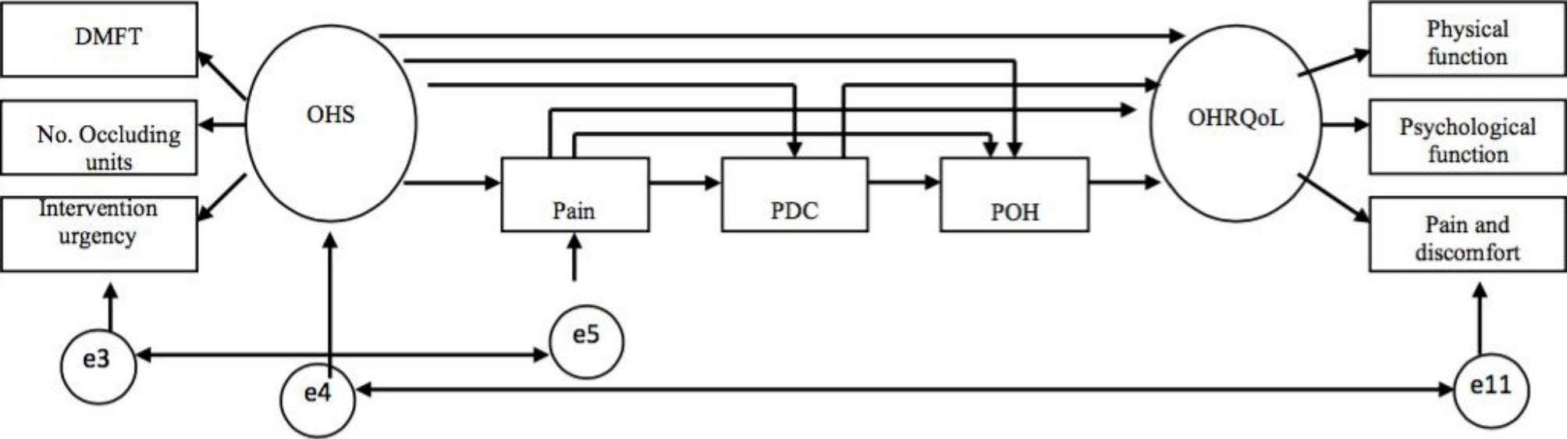
The modified Wilson and Cleary model [2] examining the direct relation between the adjacent and non-adjacent variables. Residual errors were eliminated. *OHS: Oral Health Status, PDC: Perceived Difficulty of Chewing, POH: Perceived Oral Health

Two modified Wilson and Cleary models were investigated in this study. Model 1 represented the basic Wilson and Cleary model linking oral health status to OHRQoL and their relation to the demographic and socioeconomic factors (Fig. 1). In this model, only the links between the five main adjacent levels were examined as direct paths. Model 2 examined the pathways between adjacent and non-adjacent levels without the inclusion of demographic and socioeconomic factors (Fig. 2).

The measures chosen to operationalize Wilson and Cleary model include:

#### Biological and physiological factors

presented as oral health status (OHS): a latent variable created from three indicators: DMFT, occluding units and intervention urgency. The OHS data used in this study was a part of the study that investigated the OHS among Sudanese older adults [18]. The data were collected in accordance with the criteria proposed by the World Health Organization [20]. Dentition status was recorded as Decayed (D), Missing (M), and Filled (F) Teeth, then the DMFT score was calculated for each participant. Occluding units were recorded by their numbers and denture use as 0 = 20 or more occluding teeth, 1 = 1–19 occluding teeth using dentures, 2 = 1–19 occluding teeth not using denture, 3 = no occluding teeth using denture or 4 = no occluding teeth not using denture [21].

The examiner determined the treatment needs for the participants and their urgency as: 0 = No treatment needed, 1 = Preventive or routine treatment needed, 2 = Prompt treatment including scaling needed, 3 = Immediate (urgent) treatment needed due to pain or infection of dental and/or oral origin, 4 = Referred for comprehensive evaluation or medical/dental treatment.

#### Symptom status

was assessed by asking the participants if they experienced pain/discomfort from teeth and mouth in the past 12 months. The responses were coded as 0 = No, 1 = Yes.

#### Functional status

was assessed by the perceived difficulty of chewing (PDC) [22]. The participants were asked to report difficulty of chewing of 15 common Sudanese hard and soft foods as 0 = easy-to-chew foods, and 1 = chewed with difficulty. The scores were then computed as sum score to give a maximum PDC index score, ranged 0–15.

#### General health perception

was assessed as perceived oral health (POH) as the sum of 2 questions “How would you describe the state of your teeth” and “How would you describe the state of your gums?”. The responses of the questions were 0 = poor and 1 = good.

#### Overall quality of life

was assessed as a latent variable (OHRQoL) created from the three domains of the GOHAI.

#### Demographic and socioeconomic factors

included gender, age, level of education, and occupation.

### Data analysis

Descriptive statistics (frequency and mean ±SD) were used to report the OHS, symptom status, PDC, POH and OHRQoL. Structural Equation Models (SEM) with Maximum Likelihood Estimation (ML) was used to examine the direct and indirect relationships between the observed and latent variables within the Wilson and Cleary model.

The SEM analysis was conducted with AMOS (SPSS Statistics version 23.0, SPSS Inc., Chicago, IL). Confirmatory Factor Analysis (CFA) was used to confirm the associations between the latent variables and their observed measures. As the multivariate kurtosis in both CFA and path analysis indicated a non-normal distribution, the model was estimated with 900 bias-corrected bootstrap samples. The bootstrap standardized estimates, standard errors (SE) and bias-corrected 95% confidence interval (CI) were reported.

The overall model fit was tested with chi-squared test and P-value, and a 2-index presentation strategy [23]. Low Chi-squared value, Comparative Fit Index (CFI) values > 0.95, in combination with Standardized Root Mean Squared Residual (SRMR) < 0.08 were used to indicate a good model fit [23].

## Results

A total of 249 older adults participated in this study. The mean age was 68.24 years (SD ± 6.7). Male participants represented 64.3% of the sample. The mean DMFT and PDC scores were 15.9 (SD ± 9.1) and 3.01 (SD ± 2.8), respectively. The sociodemographic and oral health characteristics of the study participants are presented in Table 1.

**Table 1:** Sociodemographic and oral health-related characteristics among Sudanese older adults (n = 249)

Variable	% (No)
Age Groups	
60–69 years	62.7 (156)
≥ 70	37.3 (93)
**Gender**	
Male	64.3 (160)
Female	35.7 (89)
**Education Level**	
No/Poor education	67.5(168)
Good/High education	32.5 (81)
**Occupational Level**	
Unemployed	30.5 (76)
Employed	69.5 (173)
**Teeth or mouth pain during the past 12 months**	
No	49 (122)
Yes	51 (127)
**Perceived oral health (POH)**	
Poor	51.8 (129)
Good	48.2 (120)
**Occluding units**	
≥ 20 teeth	43 (107)
1–19 teeth and using dentures	3.2 (8)
1–19 teeth and not using dentures	37.8 (94)
No occluding teeth but using dentures	4.4 (11)
No occluding teeth and not using dentures.	11.6 (29)
**Intervention urgency**	
No treatment need	0.8 (2)
Preventive or routine treatment	1.2 (3)
Prompt treatment	28.1 (70)
Immediate/urgent treatment	36.9 (92)
Referred for comprehensive evaluation	33 (82)

The mean GOHAI score was 53.96 (SD ± 6.31). Trouble in biting and chewing was the most commonly reported negative impact in GOHAI, while the least reported impact was limited contact with others. Physical functions’ limitation, pain and discomfort, and psychosocial functions limitations were reported by 47.0%, 45.0% and 39.0% of the participants, respectively.

**Fig. 3:**
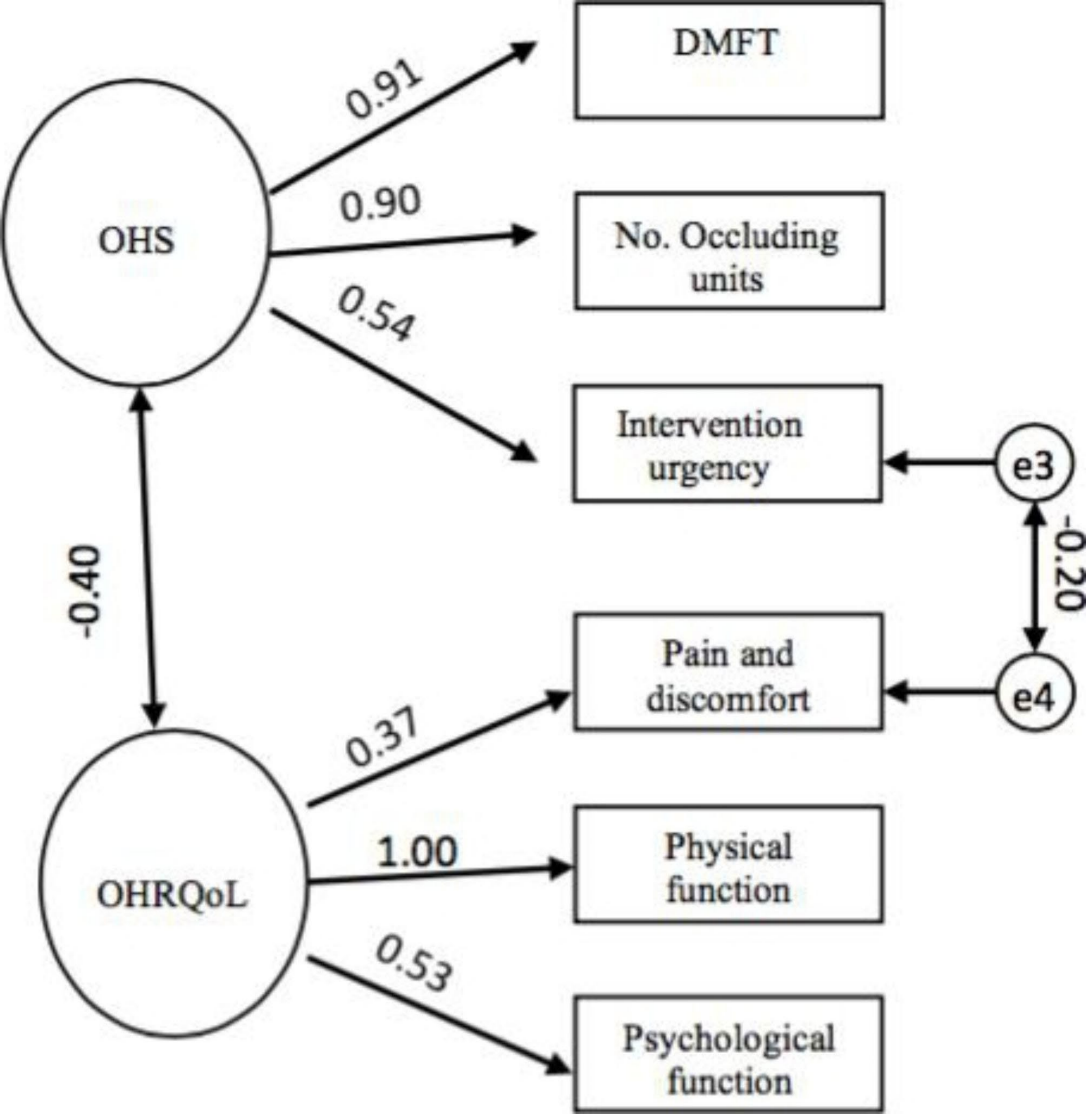
Bootstrapped Maximum Likelihood (ML) standardized estimates for Confirmatory Factor Analysis (CFA). All obtained effects were statistically significant (p < 0.05)

The CFA model and model 2 showed an acceptable fit. While the goodness of fit indices showed that model 1 did not fit the data well (Table 2). The bootstrapped estimated correlation of OHS and OHRQoL was – 0.40, exhibiting acceptable discriminant validity of the CFA. The bootstrapped standardized estimates for the model is shown in Fig. 3.

**Table 2:** Fit indices for the confirmatory factor analysis and path analysis models

Model	X^2^/d.f	P value	CFI	SRMR
**CFA**	4.4	< 0.001	**0.96**	**0.08**
**Model 1**	9.7	< 0.001	0.60	0.16
**Model 2**	**2.5**	< 0.001	**0.97**	**0.04**

CFA: Confirmatory factor analysis, χ^2^: chi-square; d.f: Degrees of freedom; CFI: Comparative Fit Index; SRMR: Standardized Root Mean Squared Residual. Figures in bold are those that meet model fitting criteria.

Models 1 and 2 accommodate for 1.1% and 0.6% of the variance of feeling tooth and gum pain, 9.4% and 49.2% in PDC, 17.9% in both models of POH and 26.1% and 74.7% in OHRQoL, respectively. The bootstrapped standardized estimates, SE and bias-corrected 95% CI of the direct and indirect effects on models 1 and 2 are presented in Tables 3 and 4. Three of the direct paths in model 1 were statistically significant with the expectation of OHS → symptom status path. Age and gender have only direct effect on OHS, while gender has indirect effect on POH. Education and occupation have direct and indirect effects on POH and OHRQoL.

In model 2, all the proposed direct effects between adjacent and non-adjacent variables were statistically significant except for three paths (OHS→pain, OHS→POH and OHS→OHRQoL). On the other hand, there was an indirect effect of OHS on OHRQoL through PDC. Feeling pain has indirect effect on POH and OHRQoL, while PDC has indirect effects on OHRQoL.

**Table 3:** Maximum likelihood bootstrapped standardized direct effects for the Wilson and Cleary model among Sudanese older adults n = 249

	Model 1			Model 2		
	ß	Bootstrap SE	Bias-corrected 95% CI	ß	Bootstrap SE	Bias-corrected 95% CI
OHS→DMFT	0.98	0.04	0.91–1.1*	0.92	0.01	0.87–0.95*
→Occluding units	0.84	0.04	0.76–0.92*	0.90	0.01	0.86–0.93*
→Intervention	0.49	0.07	0.32–0.62*	0.50	0.07	0.33–0.62*
→ Pain	-0.07	0.07	-0.20-0.08	-0.08	0.06	-0.20-0.07
→PDC	-	-	-	0.69	0.04	0.59–0.76*
→POH	-	-	-	-0.17	0.11	-0.38-0.04
→OHRQoL	-	-	-	0.05	0.08	-0.12-0.22
Pain→PDC	0.13	0.06	0.005–0.25*	0.19	0.04	0.09–0.27*
→POH	-	-	-	-0.16	0.05	-0.27- -0.05*
→OHRQoL	-	-	-	-0.18	0.06	-0.31- -0.06*
PDC →POH	-0.37	0.06	-0.48- -0.24*	-0.23	0.11	-0.42- -0.003*
→OHRQoL	-	-	-	-0.72	0.07	-0.86- -0.57*
POH→OHRQoL	0.48	0.09	0.29–0.64*	0.25	0.07	0.11–0.38*
OHRQoL→Physical	0.91	0.09	0.76–1.1*	0.83	0.04	0.74–0.91*
→Psychological	0.57	0.06	0.43–0.69*	0.58	0.05	0.45–0.67*
→Pain/Discomfort	0.43	0.07	0.29–0.58**	0.69	0.07	0.53–0.84*
Gender→OHS	0.32	0.07	0.17–0.46*	-	-	-
→Pain	0.00	0.07	-0.15-0.16	-	-	-
→PDC	0.23	0.07	0.07–0.36*	-	-	-
→POH	-0.05	0.06	-0.17-0.07	-	-	-
→OHRQoL	-0.02	0.07	-0.11-0.15	-	-	-
Age →OHS	0.18	0.07	0.03–0.32*	-	-	-
→Pain	-0.04	0.07	-0.18-0.11	-	-	-
→PDC	0.07	0.06	-0.06-0.21	-	-	-
→POH	-0.07	0.06	-0.20-0.04	-	-	-
→OHRQoL	0.04	0.06	-0.08-0.17	-	-	-
Education→OHS	-0.02	0.06	-0.16-0.104	-	-	-
→Pain	-0.05	0.06	-0.18-0.06	-	-	-
→PDC	-0.12	0.06	-0.25- -0.001	-	-	-
→POH	0.02	0.05	-0.08-0.13	-	-	-
→OHRQoL	0.16	0.06	0.04–0.28*	-	-	-
Occupation →OHS	-0.07	0.06	-0.20-0.06	-	-	-
→Pain	-0.02	0.07	-0.16-0.11	-	-	-
→PDC	-0.05	0.06	-0.18- 0.07	-	-	-
→POH	0.15	0.06	-0.28- -0.04*	-	-	-
→OHRQoL	0.09	0.06	-0.03-0.22	-	-	-

ß: bootstrapped standardized estimate; SE: Standard Error; CI: Confidence Interval, OHS: Oral Health Status, PDC: Perceived Difficulty of Chewing, POH: Perceived Oral Health, OHRQoL: Oral Health Related Quality of Life, *: P < 0.05, **: P ≤ 0.01

**Table 4:** Maximum likelihood bootstrapped standardized indirect effects for the Wilson and Cleary model among Sudanese older adults n = 249

	Model 1			Model 2		
	**ß**	**Bootstrap** **SE**	**Bias-corrected 95% CI**	**ß**	**Bootstrap** **SE**	**Bias-corrected 95% CI**
OHS→PDC →POH →OHRQoL Pain→POH →OHRQoL PDC → OHRQoL Gender→Pain →PDC →POH →OHRQoL Age →Pain →PDC →POH →OHRQoL Education→Pain →PDC →POH →OHRQoL Occupation →Pain →PDC →POH →OHRQoL	-0.01 0.003 0.002 -0.04 -0.02 -0.17 -0.02 -0.003 -0.08 -0.06 -0.01 -0.007 -0.02 -0.04 0.002 -0.007 0.05 0.03 0.005 -0.003 0.02 -0.06	0.01 0.004 0.002 0.02 0.01 0.05 0.02 0.01 0.02 0.03 0.01 0.01 0.02 0.03 0.008 0.01 0.02 0.03 0.009 0.01 0.02 0.03	-0.02-0.008 -0.003-0.01 -0.001-0.007 -0.10- -0.003* -0.06- -0.003* -0.28- -0.08* -0.07-0.02 -0.03-0.01 -0.14- -0.03* -0.14-0.004 -0.05-0.01 -0.03-0.01 -0.07-0.02 -0.12-0.01 -0.01-0.02 -0.03-0.01 0.003-0.10* -0.01-0.10 -0.005-0.03 -0.02-0.01 -0.02-0.07 -0.14- -0.01*	-0.01 -0.14 -0.55 -0.04 -0.18 -0.06 - - - - - - - - - - - - - - - -	0.01 0.07 0.06 0.02 0.04 0.03 - - - - - - - - - - - - - - - -	-0.04-0.01 -0.28-0.01 -0.69- -0.43** -0.09- -0.001* -0.26- -0.11* -0.14- -0.006* - - - - - - - - - - - - - - - -

ß: bootstrapped standardized estimate; SE: Standard Error; CI: Confidence Interval, OHS: Oral Health Status, PDC: Perceived Difficulty of Chewing, POH: Perceived Oral Health, OHRQoL: Oral Health Related Quality of Life, *: P < 0.05, **: P ≤ 0.01

## Discussion

The aim of this study was to identify the possible relations between OHS, demographic and socioeconomic predictors on OHRQoL using Wilson and Cleary model. The use of SEM in the analysis of this study allowed for the inclusion of multiple items through complex statistical methods to be able to detect both direct and indirect relations between the variables. SEM has been found to exhibit superior properties compared to regression analysis in overcoming the limitations of regression by decomposing the sources of correlation among independent variables and make it possible for each variable in a path model to be treated simultaneously as both a predictor and as an outcome [24]. Moreover, the use of a two-index strategy avoids some of the problems of sample size and distributional misspecification associated with x^2^statistics in the evaluation of the model fit [23].

The mean GOHAI score reported in this study was high, representing a relatively good OHRQoL. Participants reported more functional limitations than pain and discomfort in contrast to poor OHRQoL and physical pain and psychological discomfort reported in other studies [5, 25]. A possible explanation is that satisfaction or dissatisfaction with oral health can be influenced by different individual expectations and experiences. As individuals’ age, they are more likely to consider minor or even severe oral health problems as insignificant at this point in their lives [26]. Also differences can be due to a range of clinical variables [27], dental care [7], personal and environmental factors [12], and social support [28]. Moreover, different studies used different instruments to measure OHRQoL [5, 29].

Model 2 showed a good fit to data as well as accommodating for 74.7% of the variance of OHRQoL, which suggests that the proposed direct paths between the adjacent and non-adjacent levels of the Wilson and Cleary model has more effect on OHRQoL of the studied population than the effect of demographic and socioeconomic variables incorporated in model 1. Model 1 may require modifications to capture other factors that may be important determinants of overall QoL. These modifications include the environmental characteristics, income, geographic location and social support [12, 30]. Although model 1 did not fit the data, it showed that older women presented with worse oral health status, and worse POH indirectly through having worse OHS. Also, highly educated participants had good perceived oral health and high QoL, while working participants had poor perceived oral health and quality of life compared to non-working and retired participants. This was in accordance with another study [31], while another study found that low education and low income predicted poor OHRQoL mediated through dental clinical status [9].

The results of this study partially confirmed the Wilson and Cleary model as it confirms three out of the four and seven out of ten of the proposed direct pathways in models 1 and 2, respectively. OHS showed no direct effect on symptom status (feeling pain), POH and OHRQoL in this study. Studies tested the application of the Wilson and Cleary models on oral health reported controversial and changing relationship between the clinical status and symptom status according to the measures chosen to operationalize the model [13, 14]. A systematic review investigated Wilson and Cleary model application in chronic diseases, eleven out of 26 studies found a link between the biological factors and symptom status [24]. One explanation might be that symptom status in this study was evaluated by only one question (pain/discomfort) that did not capture all range of symptoms associated with oral diseases such as bleeding gums, food impaction or loose dentures. Furthermore, the relationship between biological and physiological factors and symptoms is complex, as physiological abnormality may not immediately produce symptoms while some symptoms may not be clinically traceable to physiologic abnormality [24]. Also, the theory of response shift may explain why the older population may report fewer impacts with poor oral health [31]. Response shift refers to changes within people regarding their internal standards, values, or conceptualization of HRQoL over time and because of the experience of ill health.

The only link between OHS and OHRQoL in this study was through PDC in model 2. Moreover, OHRQoL was affected by feeling pain, PDC, and POH. This indicates that the oral health status of the study participants did not affect their QoL unless there was some functional impairment. This is in contrast with the findings of other studies as the dental clinical status (tooth loss, number of occluding units, having denture needs and high DMFT) predicted more impact on everyday life and OHRQoL [9, 27]. It has been reported that older persons priorities their health problems if they are severe, associated with pain or functional impairments, and restrict social inclusion [32]. Moreover, adaptation to health problems, as the general belief that teeth loss and edentulism are normal part of aging process, may reduce the impact of the oral health status on OHRQoL.

One of the limitations of the study is that the sample size was calculated to estimate the prevalence of dental caries. It was not to elicit various associations; a larger sample size would have been required to establish these associations adequately. Another limitation is that the survey was conducted in health care centers, which would increase the possibility of excluding socioeconomically advantaged elderly who can afford private treatment, elderly with no health conditions, and those with limited mobility who cannot reach the location. As such, the findings of this study cannot be generalized to all older Sudanese.

## Conclusion

The OHRQoL of the studied Sudanese older adults was generally good. Functional limitation was the most commonly reported impact. The study partially confirmed Wilson and Cleary model. OHS was associated directly to PDC and indirectly to OHRQoL. Gender and age were associated with OHS while educational level was associated with OHRQoL. Future researches investigating the relationship between clinical and non-clinical factors with OHRQoL using the Wilson and Cleary model that explore the effect of the environment and social support characteristics of the individuals are needed.

## Data Availability

The datasets generated and/or analyzed during the current study are not publicly available due to confidentiality constraints but are available from the corresponding author on reasonable request.

## List of abbreviations

OHRQoL: Oral Health Related Quality of Life
GOHAI: Geriatric Oral Health Assessment Index
HRQoL: Health Related Quality of Life
OHS: Oral Health Status
PDC: Perceived Difficulty of Chewing
POH: Perceived Oral
SEM: Structural Equation Models
ML: with Maximum Likelihood Estimation
CFA: Confirmatory Factor Analysis
SE: standard errors
CI: confidence interval
CFI: Comparative Fit Index
SRMR: Standardized Root Mean Squared Residual

## References

[1] Gil-Montoya J, Ferreira de Mello AL, Barrios R, Gonzalez-Moles MA, Bravo M (2015). Oral health in the elderly patient and its impact on general well-being: a nonsystematic review. Clin Interv Aging.

[2] Locker D, Allen F (2007). What do measures of ‘oral health-related quality of life’ measure?. Community Dent Oral Epidemiol.

[3] Allen PF. Assessment of oral health related quality of life. Health Qual Life Outcomes [Internet]. 2003;1:1–8. Available from: http://www.hqlo.com/content/1/1/40. 10.1186/1477-7525-1-40 PMC201012 14514355

[4] Macentee M (2007). Quality of life as an Indicator of oral health in older people. J Am Dent Assoc.

[5] Kotzer RD, Lawrence HP, Clovis JB, Matthews DC. Oral health-related quality of life in an aging Canadian population. Health Qual Life Outcomes [Internet]. 2012;10(50):1–12. Available from: http://www.hqlo.com/content/10/1/50. 10.1186/1477-7525-10-50 PMC3480903 22587387

[6] Olofsson J, Ljungqvist O, Stjernfeldt PE, Wardh I, Olin AO. Relationships between oral health-related quality of life and objective and subjective Masticatory ability in geriatric patients: a pilot study. 2017;3(1):5.

[7] Gagliardi D, Slade G, Sanders A. Impact of dental care on oral health-related quality of life and treatment goals among elderly adults. Aust Dent J 2008 Mar;53(1):26–33. 10.1111/j.1834-7819.2007.00005.x 18304238

[8] Hernández-Palacios RD, Ramírez-Amador V, Jarillo-Soto EC, Irigoyen-Camacho ME, Mendoza-Núñez VM. Relationship between gender, income and education and self-perceived oral health among elderly Mexicans. An exploratory study. Ciênc Saúde Coletiva. 2015 Apr;20(4):997–1004. 10.1590/1413-81232015204.00702014 25923612

[9] Rebelo MAB, Cardoso EM, Robinson PG, Vettore MV (2016). Demographics, social position, dental status and oral health-related quality of life in community-dwelling older adults. Qual Life Res.

[10] Baiju R (2017). Oral health and quality of life: current concepts. J Clin Diagn Res.

[11] Short SE, Mollborn S. Social determinants and health behaviors: conceptual frames and empirical advances. Curr Opin Psychol. 2015 Oct;5:78–84. 10.1016/j.copsyc.2015.05.002 PMC4511598 26213711

[12] Wilson IB, Cleary P (1995). Linking clinical variables with health-related quality of life. A conceptual model of patient outcomes. J Am Med Assoc.

[13] Baker S, Pankhurst C, Robinson PG (2007). Testing relationships between clinical and non-clinical variables in xerostomia: a structural equation model of oral health-related quality of life. Qual Life Res.

[14] Dos Santos C, Celeste R, Hilgert J, Hugo F. Testing the applicability of a model of oral health-related quality of life. Cad Saúde Pública [Internet]. 2015;31(9):1871–80. Available from: 10.1590/0102-311X00119914. 10.1590/0102-311X00119914 26578012

[15] GBD 2017 Oral Disorders Collaborators (2020). Global, Regional, and national levels and Trends in Burden of oral conditions from 1990 to 2017: a systematic analysis for the global burden of Disease 2017 study. J Dent Res.

[16] Petersen P, Baez R, Ogawa H (2020). Global application of oral disease prevention and health promotion as measured 10 years after the 2007 World Health Assembly statement on oral health. Community Dent Oral Epidemiol.

[17] Khalifa N, Allen PF, Abu-bakr NH, Abdel-Rahman ME, Abdelghafar KO. A survey of oral health in a sudanese population. BMC Oral Health. 2012;12(1). 10.1186/1472-6831-12-5 PMC3311612 22364514

[18] Salih M, Ali R, Nasir E (2022). Oral health status and associated factors among sudanese older adults: a cross-sectional study. Gerodontology.

[19] Atieh M (2008). Arabic version of geriatric oral health assessment index. Gerodontology.

[20] WHO. Oral Health Surveys Basic Methods. 5TH ed. WHO Press; 2013.

[21] Shimazaki Y, Soh I, Saito T, Yamashita Y, Koga T, Miyazaki H, et al. Influence of Dentition Status on Physical Disability, Mental Impairment, and Mortality in Institutionalized Elderly People. J Dent Res. 2001 Jan;80(1):340–5. 10.1177/00220345010800010801 11269726

[22] Khalifa N, Allen F, Abu-bakr PH, Abdel-Rahman NE (2013). Chewing ability and associated factors in a sudanese population. J Oral Sci.

[23] Hu L, Bentler PM (1999). Cutoff criteria for fit indexes in covariance structure analysis: conventional criteria versus new alternatives. Struct Equ Model Multidiscip J.

[24] Ojelabi A, Graham Y, Haighton H, Ling J. A systematic review of the application of Wilson and Cleary health-related quality of life model in chronic diseases. Health Qual Life Outcomes. 2017;15(241). 10.1186/s12955-017-0818-2 PMC5725920 29228977

[25] Cornejo M, Perez G, de Kc L, Casals-Peidro E, Borrell C (2013). Oral health-related quality of life in institutionalized elderly in Barcelona (Spain). Med Oral Patol Oral Cirugia Bucal.

[26] Machado V, Botelho J, Proenca L, Alves R, Oliveira MJ, Aguas A et al. Periodontal status, perceived stress, diabetes mellitus and oral hygiene care on quality of life: a structural equation modelling analysis. BMC Oral Health [Internet]. 2020;20(229):1–11. Available from: 10.1186/s12903-020-01219-y. 10.1186/s12903-020-01219-y PMC7441730 32819351

[27] Gerritsen A, Allen P, Witter D, Bronkhorst E, Creugers N. Tooth loss and oral health-related quality of life: a systematic review and meta-analysis. Health Qual Life Outcomes. 2010;8(126). 10.1186/1477-7525-8-126 PMC2992503 21050499

[28] Niesten D, Witter D, Bronkhorst E, Creugers N. Oral health-related quality of life and associated factors in a care-dependent and a care-independent older population. J Dent [Internet]. 2016 Dec [cited 2019 Feb 11];55:33–9. Available from: https://linkinghub.elsevier.com/retrieve/pii/S030057121630183X. 10.1016/j.jdent.2016.09.007 27662794

[29] Botelho J, Machado V, Proença L, Oliveira MJ, Cavacas M, Amaro L et al. Perceived xerostomia, stress and periodontal status impact on elderly oral health-related quality of life: findings from a cross-sectional survey. BMC Oral Health [Internet]. 2020;20(199):1–9. Available from: 10.1186/s12903-020-01183-7. 10.1186/s12903-020-01183-7 PMC7350690 32650751

[30] Ekback G, Astrom A, Klock K, Ordell S, Lennart U (2012). Oral health of 65-year olds in Sweden and Norway: a global question and ICF, the latest conceptual model from WHO. Acta Odontol Scand.

[31] Masood M, Newton T, Bakri N, Khalid T, Masood Y (2017). The relationship between oral health and oral health related quality of life among elderly people in United Kingdom. J Dent.

[32] Junius-Walker U, Schleef T, Vogelsang U, Dierks ML. How older patients priorities their multiple health problems: a qualitative study. BMC Geriatr. 2019;19(362):1–8. Available from: https://bmcgeriatr.biomedcentral.com/articles/10.1186/s12877-019-1373-y. 10.1186/s12877-019-1373-y PMC6925512 31864309

